# Aging‐associated decrease of PGC‐1α promotes pain chronification

**DOI:** 10.1111/acel.14177

**Published:** 2024-05-17

**Authors:** Xinbo Wu, Liuyue Yang, Zihua Li, Chenzheng Gu, Kaiyan Jin, Andrew Luo, Nabeel Faiyaz Rasheed, Isabella Fiutak, Kristina Chao, Amy Chen, Jianren Mao, Qian Chen, Weihua Ding, Shiqian Shen

**Affiliations:** ^1^ Department of Anesthesia, Critical Care and Pain Medicine Massachusetts General Hospital, Harvard Medical School Boston Massachusetts USA; ^2^ University of Missouri‐Kansas City School of Medicine Kansas City Missouri USA; ^3^ The Winsor School Boston Massachusetts USA; ^4^ Summer Intern Program Massachusetts General Hospital, Harvard Medical School Boston Massachusetts USA; ^5^ Chinese Academy of Sciences Zhongshan Institute for Drug Discovery, Shanghai Institute of Materia Medica Shanghai China; ^6^ Present address: Shanghai 10th Hospital Tongji University School of Medicine Shanghai China

**Keywords:** aging, mice, pain, PGC‐1α, somatosensory cortex

## Abstract

Aging is generally associated with declining somatosensory function, which seems at odds with the high prevalence of chronic pain in older people. This discrepancy is partly related to the high prevalence of degenerative diseases such as osteoarthritis in older people. However, whether aging alters pain processing in the primary somatosensory cortex (S1), and if so, whether it promotes pain chronification is largely unknown. Herein, we report that older mice displayed prolonged nociceptive behavior following nerve injury when compared with mature adult mice. The expression of peroxisome proliferator‐activated receptor‐gamma coactivator‐1α (PGC‐1α) in S1 was decreased in older mice, whereas PGC‐1α haploinsufficiency promoted prolonged nociceptive behavior after nerve injury. Both aging and PGC‐1α haploinsufficiency led to abnormal S1 neural dynamics, revealed by intravital two‐photon calcium imaging. Manipulating S1 neural dynamics affected nociceptive behavior after nerve injury: chemogenetic inhibition of S1 interneurons aggravated nociceptive behavior in naive mice; chemogenetic activation of S1 interneurons alleviated nociceptive behavior in older mice. More interestingly, adeno‐associated virus‐mediated expression of PGC‐1α in S1 interneurons ameliorated aging‐associated chronification of nociceptive behavior as well as aging‐related S1 neural dynamic changes. Taken together, our results showed that aging‐associated decrease of PGC‐1α promotes pain chronification, which might be harnessed to alleviate the burden of chronic pain in older individuals.

## Graphical abstract

Our study reveals that PGC‐1α is implicated in prolonged nociceptive‐like behavior in older mice. We demonstrate that decreased PGC‐1α levels in the somatosensory cortex contribute to pain chronification, fueled by dampened cortical interneuron activity and heightened excitatory neuronal activity. These results shed light on the mechanisms underlying aging‐related pain chronification.

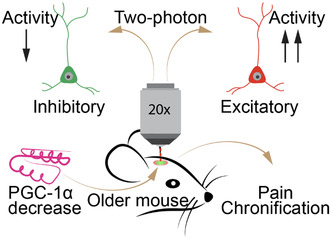

**Abbreviations**
CCI: chronic contriction injury of sciatic nerve
DREADDs: designer receptors exclusively activated by disigner drugs
GABA: gamma‐aminobutyric acid
PGC‐1α: peroxisome proliferator‐activated receptor‐gamma coactivator‐1α
S1: promary somatosensory cortex
S1HL: Hindlimb region of primary somatosensory cortex

## 1 INTRODUCTION

Aging is accompanied by gradual, yet significant, physiological changes in all organ systems, including the nervous system. For example, main sensory modalities including vision, hearing, smell, and taste are generally in decline with advanced age (Cavazzana et al., 2018; Schumm et al., 2009). Additionally, advanced age is associated with declining somatosensation, evidenced by increased mechanical, vibratory, cold, and warmth detection thresholds (Dunn et al., 2015; Johnson et al., 2021; Low Choy et al., 2007). Chronic pain, which has a key somatosensory domain, is highly prevalent in older individuals (Thornbury & Mistretta, 1981; Wang et al., 2006). Notably, age‐related decline in somatosensation seems at odds with the high prevalence of chronic pain in older adults. This discrepancy can be explained, in part, by the high prevalence of degenerative diseases such as osteoarthritis in older people (Marks, 2018; Zhang et al., 2019). These age‐related degenerative diseases provide peripheral inputs to the nociceptive system underlying chronic pain. However, it is unclear if central pain processing mechanisms also contribute to the high prevalence of chronic pain in older individuals.

The primary somatosensory cortex (S1) is a critical brain region for the processing of somatosensory information, which has been implicated in pain perception (Bushnell et al., 1999; Vierck et al., 2013). For example, in a mouse model of trigeminal neuralgia (Ding, Fischer, et al., 2023), S1 regional neural dynamics were pushed into a synchronized state, which was indispensable for spontaneous pain‐like behavior (Ding, Yang, et al., 2023). Neuropathic pain was found to be linked to S1 somatostatin interneuron dysfunction. Activation of these interneurons prevented the development of neuropathic pain (Cichon et al., 2017). In a mouse model of inflammatory pain, S1 was found to have increased neuronal activity and connectivity (Okada et al., 2021). In humans, postherpetic neuralgia was associated with S1 plasticity, shown as functional reorganization in magnetic resonance imaging, implicated in both evoked and spontaneous pain intensities (Li et al., 2022). Successful treatment of carpal tunnel syndrome using acupuncture was linked to the rewiring of the S1 (Maeda et al., 2017).

Despite the prominent role of S1 in pain processing, its potential role in age‐related changes in pain processing has only begun to unravel (Yezierski, 2012). Using resting‐state functional connectivity analysis of magnetic resonance imaging, a recent study found that older, as compared to younger adults, displayed higher pain thresholds, enhanced functional connectivity of somatosensory cortices, and reduced connectivity between regions involved in pain inhibition (Gonzalez‐Roldan et al., 2020). Particularly, older participants showed reduced functional connectivity between key nodes of the descending pain inhibitory pathway (Gonzalez‐Roldan et al., 2020). These results highlight a plausible role for the central nervous system in the greater vulnerability to chronic pain in older adults. In line with this, older mice displayed exaggerated nociceptive behavior in response to tonic painful stimuli such as acetone and capsaicin (Millecamps et al., 2020). Using a monoiodoacetate‐induced knee osteoarthritis model, acute pain processing was similar between young and older rats. Hyperalgesia‐like nociceptive behavior lasted significantly longer in older rats than young adults (Ro et al., 2020). This model of aging‐related pain chronification was related to changes in the pain modulatory network in older animals (Da Silva et al., 2021).

Peroxisome Proliferator‐Activated Receptor Gamma Coactivator 1‐alpha (PGC‐1α) serves as a crucial transcriptional coactivator, exerting control over mitochondrial biogenesis, energy metabolism, and neuronal function (Baur et al., 2006; Liu et al., 2007; Sahin et al., 2011). These processes are integral to pain modulation and processing within the somatosensory cortex. Recent research indicates that PGC‐1α may play a role in modulating pain sensitivity and the development of chronic pain conditions (Miao, Adkins‐Threats, et al., 2020). It is implicated in regulating the expression of genes involved in nociception, neuroinflammation, and synaptic transmission within pain pathways. Furthermore, PGC‐1α influences neuronal excitability and synaptic plasticity in regions like the dorsal horn of the spinal cord and the somatosensory cortex (Cheng et al., 2012; Vanaveski et al., 2021), implicated in pain processing. Changes in PGC‐1α expression or activity have been associated with alterations in pain behavior in animal models of neuropathic and inflammatory pain. While upregulation of PGC‐1α is linked to reduced pain sensitivity, downregulation or dysfunction may worsen pain symptoms (Miao, Adkins‐Threats, et al., 2020). However, the specific influence of cortical PGC‐1α on pain in older individuals and its role in promoting pain chronification remains largely unexplored.

In this study, we examined the role of S1 in aging‐related pain chronification by studying nociceptive behavior and S1 neural dynamics following chronic constriction injury of the sciatic nerve (CCI) in mature adult and older mice. Our results suggest that nerve injury in older mice induced prolonged nociceptive behavior and sustained abnormal S1 neural dynamics, revealed by intravital two‐photon calcium imaging. Manipulating S1 neural dynamics using chemogenetics influenced nociceptive behavior after nerve injury. Mechanistically, aging was accompanied by decreased expression of PGC‐1α in S1. Adeno‐associated virus‐mediated expression of PGC‐1α in S1 interneurons ameliorated aging‐related pain chronification. Taken together, our results reveal a cortical mechanism underlying aging‐related pain chronification that could be harnessed for effective pain treatment in older adults.

## 2 RESULTS

### 2.1 Age‐related differences in nociceptive behavior following chronic constriction injury in mice

**FIGURE 1:**
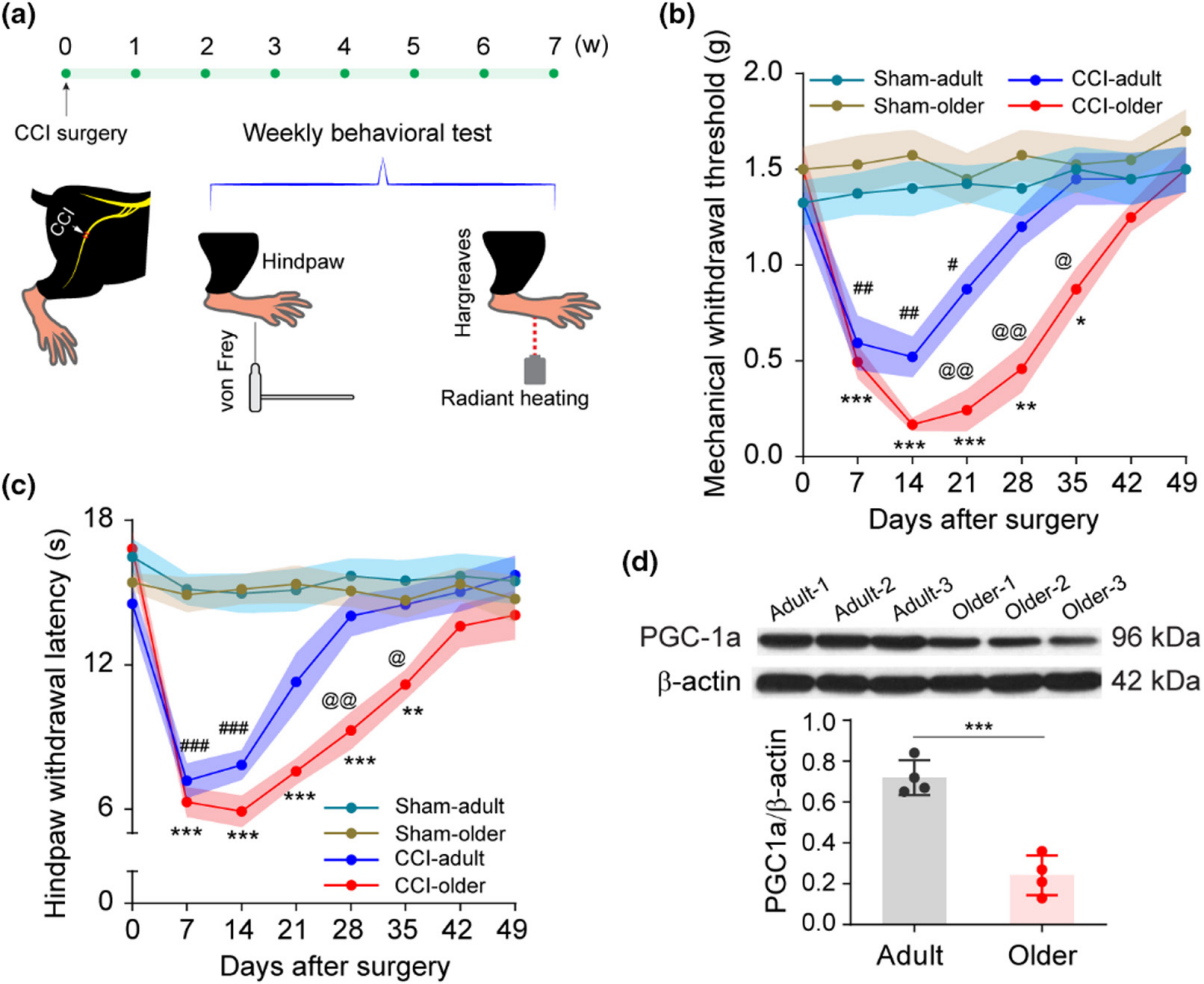
Aging is associated with pain chronification. (a–c) Chronic constriction injury of sciatic nerve (CCI) results in prolonged pain behavior in older mice. (a) Diagram and sketch depict CCI surgery and behavioral tests. Both mechanical withdrawal threshold and hindpaw withdrawal latency were applied before CCI surgery and weekly for 7 weeks after CCI surgery. (b) Mechanical withdrawal threshold and (c) Hindpaw withdrawal latency showed significant different pain behaviors at indicated time points. *N* = 8 mice (male 4 mice, female 4 mice) each group, data are presented as mean ± SEM, two‐way ANOVA followed by Bonferroni post hoc test was performed for the comparison among groups. Sham‐older versus CCI‐older *p < 0.05, **p < 0.01, ***p < 0.001; Sham‐adult versus CCI‐adult ^#^ *p* < 0.05, ^##^ *p* < 0.01, ^###^ *p* < 0.001; CCI‐adult versus CCI‐older ^@^ *p* < 0.05, ^@@^ *p* < 0.01. (d) Western blot experiment was carried out in naïve adult and older mice, and it shows peroxisome proliferator‐activated receptor gamma coactivator 1‐alpha (PGC‐1α) protein deficiency in older mice. Statistics indicated significant difference of PGC‐1α/β‐Actin between adult and older mice. *N* = 4, data are presented as mean ± SD, two‐tailed unpaired *t* test, adult versus older***p < 0.001.

Mature adult (4‐month‐old adult) and older (18‐months‐old) C57BL/6 mice (*N* = 8 mice) underwent chronic constriction injury to the sciatic nerve (CCI) followed by testing for nociceptive behavior, including hindpaw mechanical and thermal withdrawal thresholds (Figure 1a). Older mice displayed slightly higher, albeit not statistically significant, baseline withdrawal parameters compared with mature adults. After CCI, mature adults decreased their withdrawal thresholds, which returned to pre‐injury baseline levels around 4 weeks post‐injury. However, older mice displayed significantly lower withdrawal parameters than adult mice starting 3 weeks post‐injury, which then gradually returned to baseline levels at 7 weeks post‐nerve injury (Figure 1b,c). Therefore, older mice displayed prolonged nociceptive behaviors, referred to as ‘aging‐associated pain chronification’. Additionally, our data did not reveal significant disparities between the adult male and female mice (Figure S1a,b).

Previously, our lab reported that PGC‐1α (peroxisome proliferator‐activated receptor‐gamma coactivator‐1a), a transcriptional coactivator that regulates mitochondria biogenesis and neuronal function (St‐Pierre et al., 2006), was implicated in prolonged nociceptive behavior after burn injury. When PGC‐1α protein expression in S1 was examined in naïve animals (*n* = 3 mice), older mice displayed significantly lower levels than mature adult mice (Figure 1d). The aging‐related decrease of PGC‐1α in S1 is consistent with reports implicating PGC‐1α in aging‐related muscle wasting and neurodegeneration (Anderson & Prolla, 2009; Katsouri et al., 2016; Qin et al., 2009).

### 2.2 PGC‐1α haploinsufficiency is associated with prolonged nociceptive behavior and sustained cortical hyperactivation following nerve injury

**FIGURE 2:**
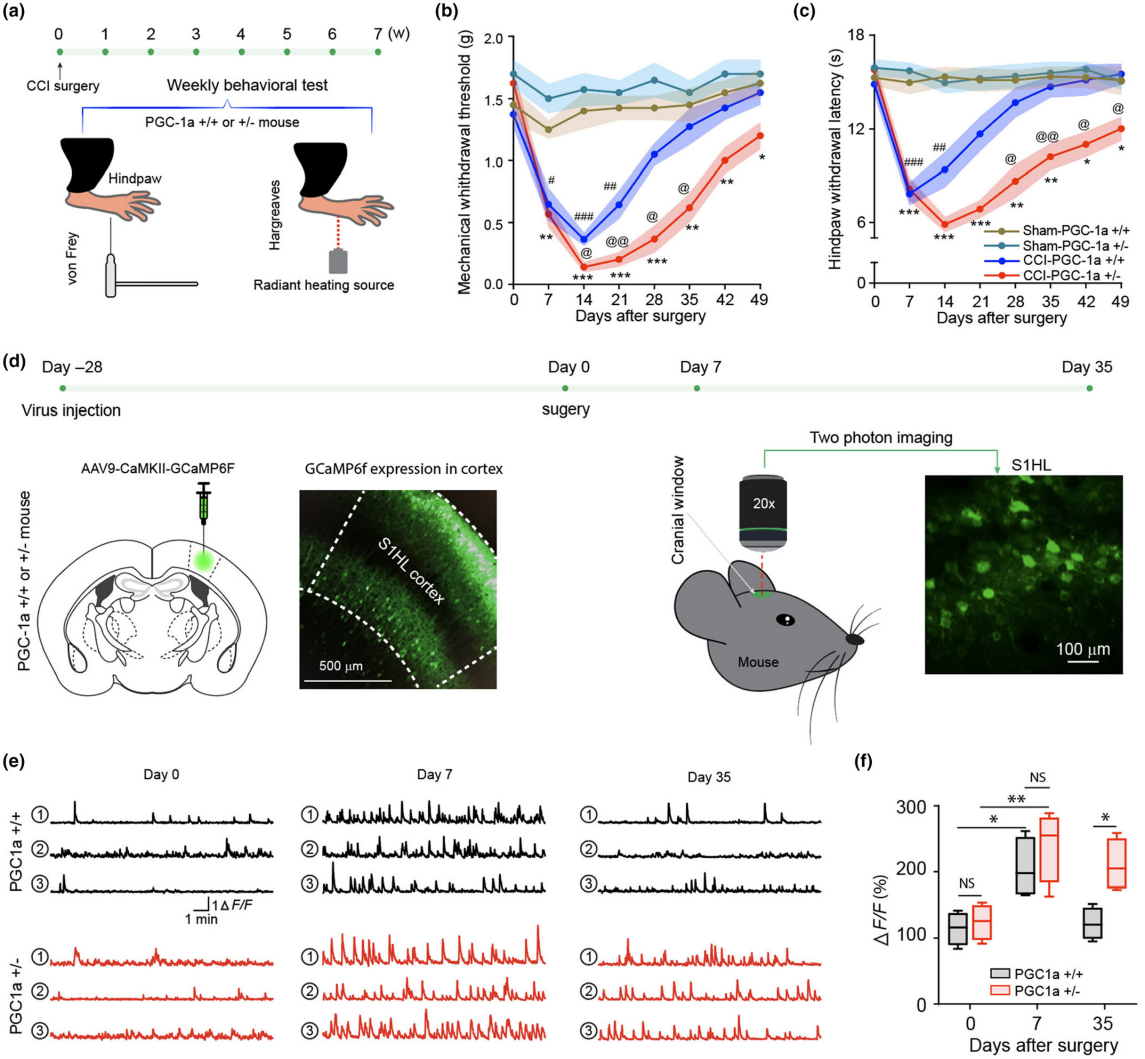
PGC‐1α haploinsufficiency leads to pain chronification and altered S1 neural dynamics. (a–c) CCI surgery induced prolonged pain behaviors in PGC‐1α (+/−) but not PGC‐1α (+/+) mice. (a) Diagram and sketch depict experiment design. (b) Mechanical withdrawal threshold and (c) Hindpaw withdrawal latency. *N* = 8 mice (4 male mice, 4 female mice) each group, data are presented as mean ± SEM, two‐way ANOVA test followed by Bonferroni post hoc test was performed for the comparison among groups. Sham‐PGC‐1α (+/−) versus CCI‐PGC‐1α (+/−) *p < 0.05, **p < 0.01, ***p < 0.001; sham‐PGC‐1α (+/+) versus CCI‐PGC‐1α (+/+) ^#^ *p* < 0.05, ^##^ *p* < 0.01, ^###^ *p* < 0.001; CCI‐PGC‐1α (+/−) versus CCI‐PGC‐1α (+/+) ^@^ *p* < 0.05, ^@@^ *p* < 0.01. (d–f) PGC‐1α deficiency results in neural hyperactivity in S1HL cortex of PGC‐1α (+/−) mice. (d) Diagram and sketch depict experiment design. AAV9‐CaMKII‐GCaMP6f virus was microinjected into S1HL cortex and a representative image of brain slice shows virus expression in S1HL cortex. After 4 weeks of viral transfection, intravital two‐photon was performed before CCI surgery and the same imaging view was followed up at 7 and 35 days after CCI surgery. A representative image on the right shows calcium signal under two‐photon microscope. (e) Three sample traces of excitatory neuron activity in each group before CCI surgery (day 0) and day 7, 35 days after CCI surgery. (f) Integrated calcium signal ΔF/F. Excitatory neuronal activities in S1HL cortex of both PGC‐1α (+/−) and PGC‐1α (+/+) mice were significantly increased after CCI surgery at day 7 comparing with baseline at day 0, and ΔF/F was statistically different at day 35 between PGC‐1α (+/−) and PGC‐1α (+/+) mice. *N* = 4 mice (male), box represents 25th, 50th, and 75th percentile and whisker represent minimum/maximum values. Two‐way ANOVA followed by Bonferroni post hoc test was performed for the comparison between groups. *p < 0.05; **p < 0.01; NS: not significant (*p* > 0.05).

To examine if decreased levels of PGC‐1α could facilitate pain chronification, PGC‐1α +/− (4 months old, heterozygous) and PGC‐1α +/+ (4 months old, wild‐type littermates, *N* = 8 mice) were used (Figure 2a). PGC‐1a −/− (homozygous “knockout”) mice die prematurely with severe neurodegeneration; hence not used for this study. After CCI injury, wild‐type control mice resolved mechanical and thermal withdrawal parameters roughly around 4–5 weeks; PGC‐1α +/− mice failed to resolve nociceptive behaviors within 7 weeks (Figure 2b,c), similar to previously observed prolonged nociceptive behavior in older mice. S1 neural dynamics are key cortical substrates mediating nociceptive behavior (Cichon et al., 2017; Okada et al., 2021). The hindlimb (HL) region of S1 was investigated using intravital two‐photon calcium imaging (*N* = 4 mice). AAV9‐CaMKII‐GCaMP6f was microinjected to the contralateral side of nerve injury, and S1HL neuronal activities were imaged in resting state without anesthesia (Figure 2d). Results showed that at day 7 post‐nerve injury, both wild‐type PGC‐1α +/+ and heterozygous PGC‐1α +/− mice displayed enhanced calcium dynamics, consistent with increased neuronal activities in responses to nerve injury. However, at day 35‐post injury, heterozygous PGC‐1α +/− mice but not wild‐type PGC‐1α +/+ displayed enhanced calcium dynamics (Figure 2e,f). Notably, at that time point, wild‐type PGC‐1α +/+ mice completely resolved nociceptive behavior, whereas PGC‐1α +/− mice continued to display robust nociceptive behavior. As such, PGC‐1α haploinsufficiency was associated with prolonged nociceptive behavior and sustained S1 hyperactivation. Additionally, when sexual differences were examined, our data did not show significant disparities between PGC‐1a +/+ or PGC‐1a +/− male and female mice (Figure S2a,b).

### 2.3 Altered S1 neuronal dynamics in aging‐associated pain chronification

**FIGURE 3:**
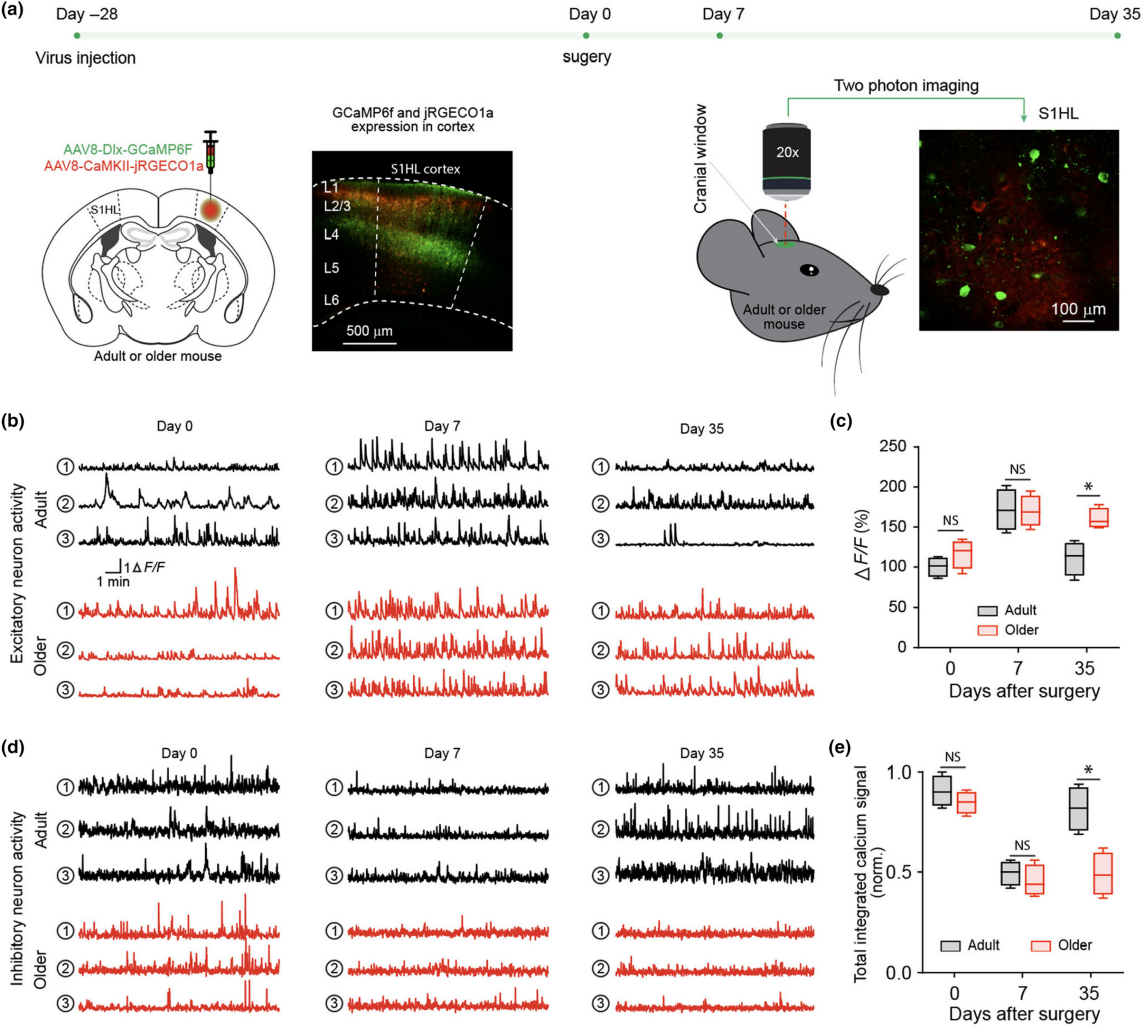
Aging‐associated pain chronification is accompanied by excitatory neuron hyperactivity and interneuron hypoactivity. (a) Diagram and sketch depict experiment design. Mixed AAV8‐CaMKII‐jRGECO1a and AAV8‐Dlx‐GCaMP6f was injected into the S1HL cortex of adult and older mice. a representative image of brain slice showed the viruses transfection in S1HL cortex. Intravital two‐photon imaging was performed before CCI surgery and day 7, 35 after CCI surgery. A representative view of calcium signal under microscope. (b) Three representative neural activity traces of excitatory neuron in each group at indicated time points. (c) Integrated calcium signal ΔF/F. *N* = 4, box represents 25th, 50th, and 75th percentile and whisker represent minimum/maximum values. Two‐way ANOVA followed by Bonferroni post hoc test was performed for the comparison between groups. *p < 0.05; NS: not significant (*p* > 0.05). (d) Three representative neural traces of inhibitory neuron in each group at indicated time points. (e) Integrated calcium signal ΔF/F. *N* = 4, box represents 25th, 50th and 75th percentile and whisker represent minimum/maximum values. Two‐way ANOVA followed by Bonferroni post hoc test was performed for the comparison between groups. *p < 0.05; NS: not significant (*p* > 0.05).

To examine S1 neuronal dynamics in aging‐associated pain chronification, two‐color intravital two‐photon calcium imaging was established to simultaneously investigate excitatory neurons and interneurons. A widely accepted viral strategy based on Dlx promoter was used for interneuron targeting (Dimidschstein et al., 2016). Previously, we reported efficient pan‐interneuron targeting using this strategy in both S1 and the hippocampus (Ding, Fischer, et al., 2023; Zhou et al., 2023). AAV8‐Dlx‐GCaMP6f (green color) and AAV8‐CaMKII‐jRGECO1a (red color) were used to image interneuron and excitatory neurons (Figure 3a), respectively, in mature adult and older mice at resting state without anesthesia (*N* = 4 mice). For excitatory neurons, at day 7 post‐nerve injury, both mature adult and older mice displayed exaggerated calcium dynamics when compared with day 0 (Figure 3b). Excitatory neuron hyperactivities largely resolved by day 35 in adult but not older mice (Figure 3c). For interneurons, at day 7 post‐nerve injury, both adult and older mice displayed muted calcium dynamics when compared with day 0 (Figure 3d). Interneuron hypoactivities largely resolved day 35 in adult but not older mice (Figure 3e). Importantly, at day 35, mature adult but not older mice were able to resolve nociceptive behavior following nerve injury. As such, aging‐related pain chronification was accompanied by sustained abnormal S1 neuron dynamics characterized by exaggerated excitatory neuron activities and muted interneuron activities. Therefore, it is plausible that altered S1 neural dynamics were responsible for aging‐related pain chronification.

### 2.4 Interrogating S1 neural dynamics in pain processing through chemogenetic manipulation

**FIGURE 4:**
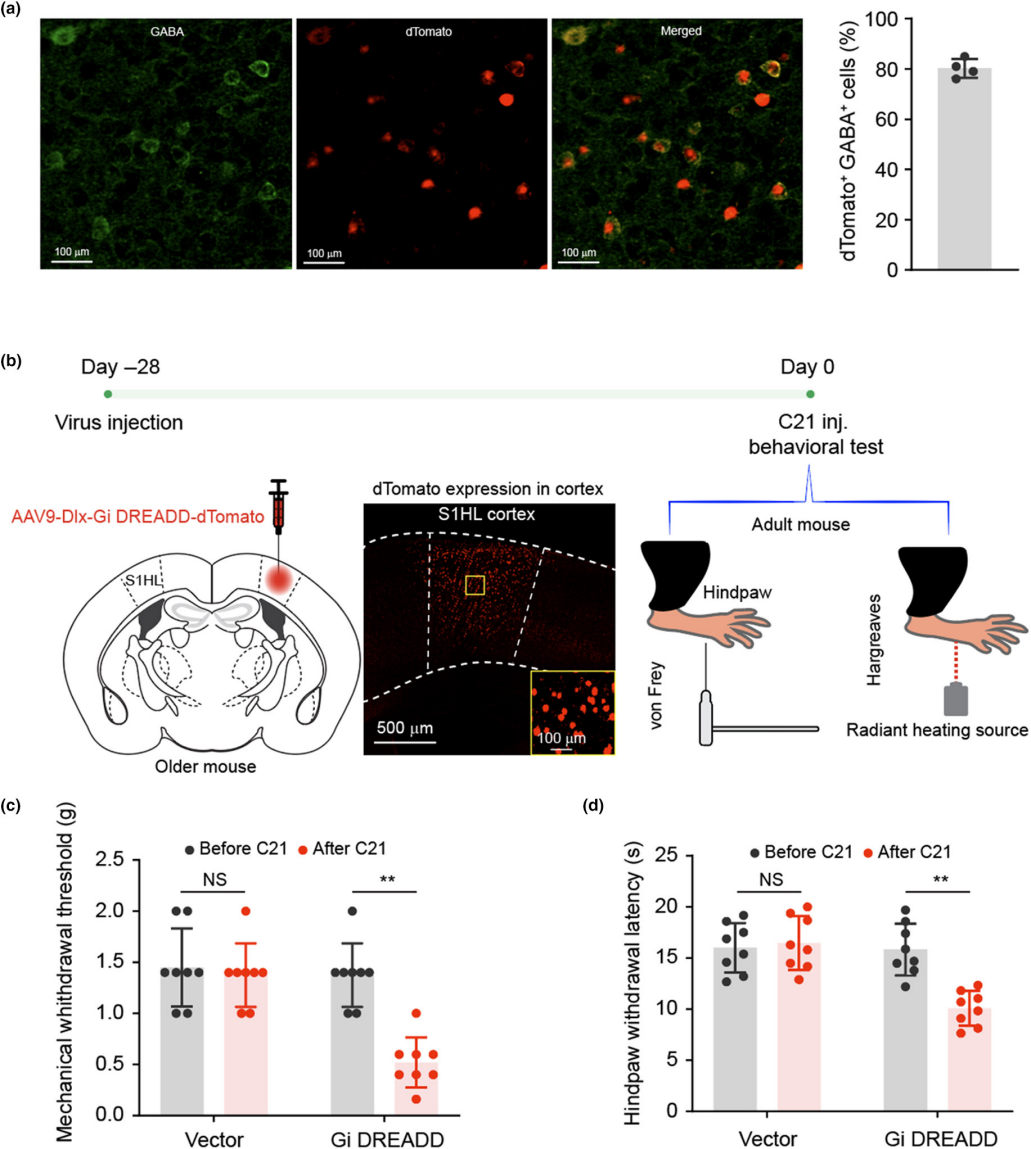
Inhibition of interneuron activity in S1HL cortex results in hyperalgesia in older mice. (a) Representative immunostaining images. Brain slices throughout S1HL cortex were selected and stained with anti‐GABA receptor. Immunofluorescence imaging showed 80% dTomato+ cells were GABA+ cells in S1HL cortex. *N* = 4, data are represented as mean ± SD. (b) Diagram and sketch depict experiment design. AAV9‐Dlx‐Gi DREADD‐dTomato was injected into S1HL of adult mice. A representative image of brain slice with box region zoomed verified viral transfection in S1HL cortex. (c) Mechanical withdrawal threshold was significantly decreased after C21 administrated. (d) Hindpaw withdrawal latency in response to S1HL inhibition. Comparing with the mice subject to vector virus, mice injected Gi GREADDs exhibited significant decreasing in hindpaw withdrawal latency after C21 administration. *N* = 8, data are presented as mean ± SD, two‐tailed paired *t* test, **p < 0.01, NS: not significant (*p* > 0.05).

**FIGURE 5:**
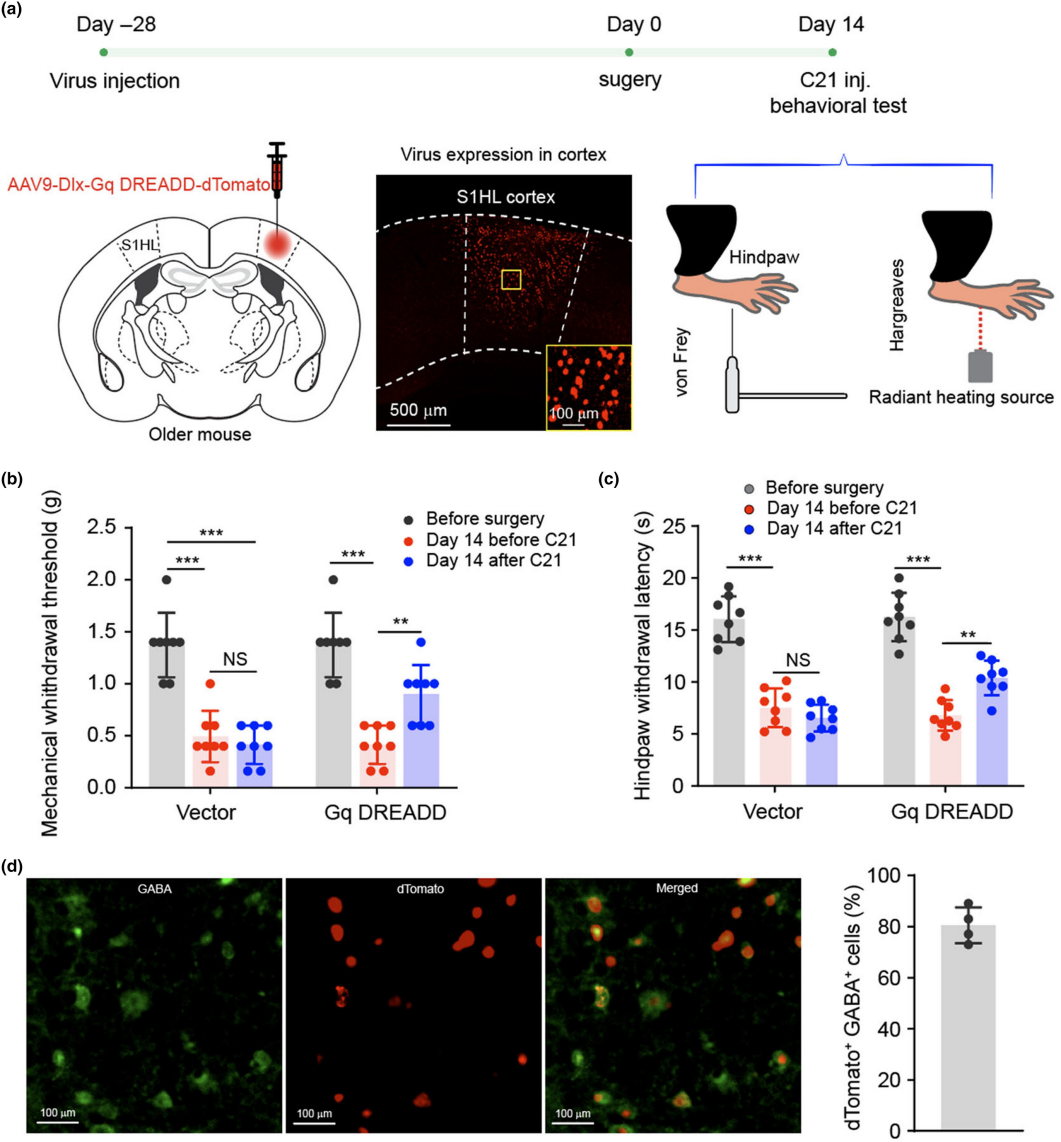
Activation of interneurons in S1HL alleviates aging‐associated pain chronification. (a) Diagram and sketch depict experimental design. AAV9‐Dlx‐Gq DREADD‐dTomato was injected into S1HL of adult mice. Following baseline behavioral test, mice received CCI surgery and C21 was administrated at day 14 after surgery. Pain behaviors were assessed 30 min after C21. (b) Mechanical withdrawal threshold. *N* = 8 mice, in mice received vector virus, before versus after surgery ***p < 0.001, before versus after C21 ***p < 0.001; in mice received Gq DREADD, before versus after surgery ***p < 0.001, before versus after C21 **p < 0.01. (c) Hindpaw withdrawal latency. Comparing with mice subjected to vector virus, mice received Gq DREADD injection in S1HL and C21 administration exhibited significant increasing in mechanical withdrawal threshold and hindpaw withdrawal latency. *N* = 8, data are presented as mean ± SD in panel B. One‐way ANOVA followed by Tukey post hoc test was performed for the comparison. **p < 0.01, ***p < 0.001. NS: not significant (*p* > 0.05). (d) Representative immunofluorescent images. Brain slices throughout S1HL cortex were selected and stained with anti‐GABA. Immunofluorescence imaging showed 80% dTomato+ cells were GABA^+^ cells in S1HL cortex. *N* = 4, data are represented as mean ± SD.

To directly interrogate S1 neural dynamics in pain processing, chemogenetic tools based on inhibitory and excitatory DREADDs (designer receptors exclusively activated by designer drugs) were adopted (Roth, 2016). S1 excitatory neurons are regulated locally by GABAergic interneurons (Chen et al., 2020). Chemogenetic targeting S1HL interneurons using AAV9‐Dlx‐Gi DREADDs showed that more than 80% GAGBergic neurons were targeted (Figure 4). C21, a DREADD agonist with no in vivo conversion to olanzapine, was used as actuator for DREADDs manipulation (Bonaventura et al., 2019). When S1 interneurons were inhibited in naïve older mice (*N* = 8 mice, Figure 4b, Figure S3), both mechanical withdrawal threshold and thermal withdrawal latency of contralateral hindpaw were decreased (Figure 4c,d), suggesting chemogenetic inhibition of interneurons induces nociceptive behavior. Conversely, in older mice after nerve injury, when interneurons were activated (*n* = 8 mice, Figure 5a), nociceptive behavior was significantly alleviated, suggesting activation of interneurons alleviates nociceptive behavior (Figure 5b,c). Subsequent immunofluorescent imaging validated that over 80% neurons expressing Gq DEADD were GABAergic interneurons (Figure 5d, Figure S4).

### 2.5 Modulating PGC‐1α levels in S1 interneurons alleviates aging‐related pain chronification

**FIGURE 6:**
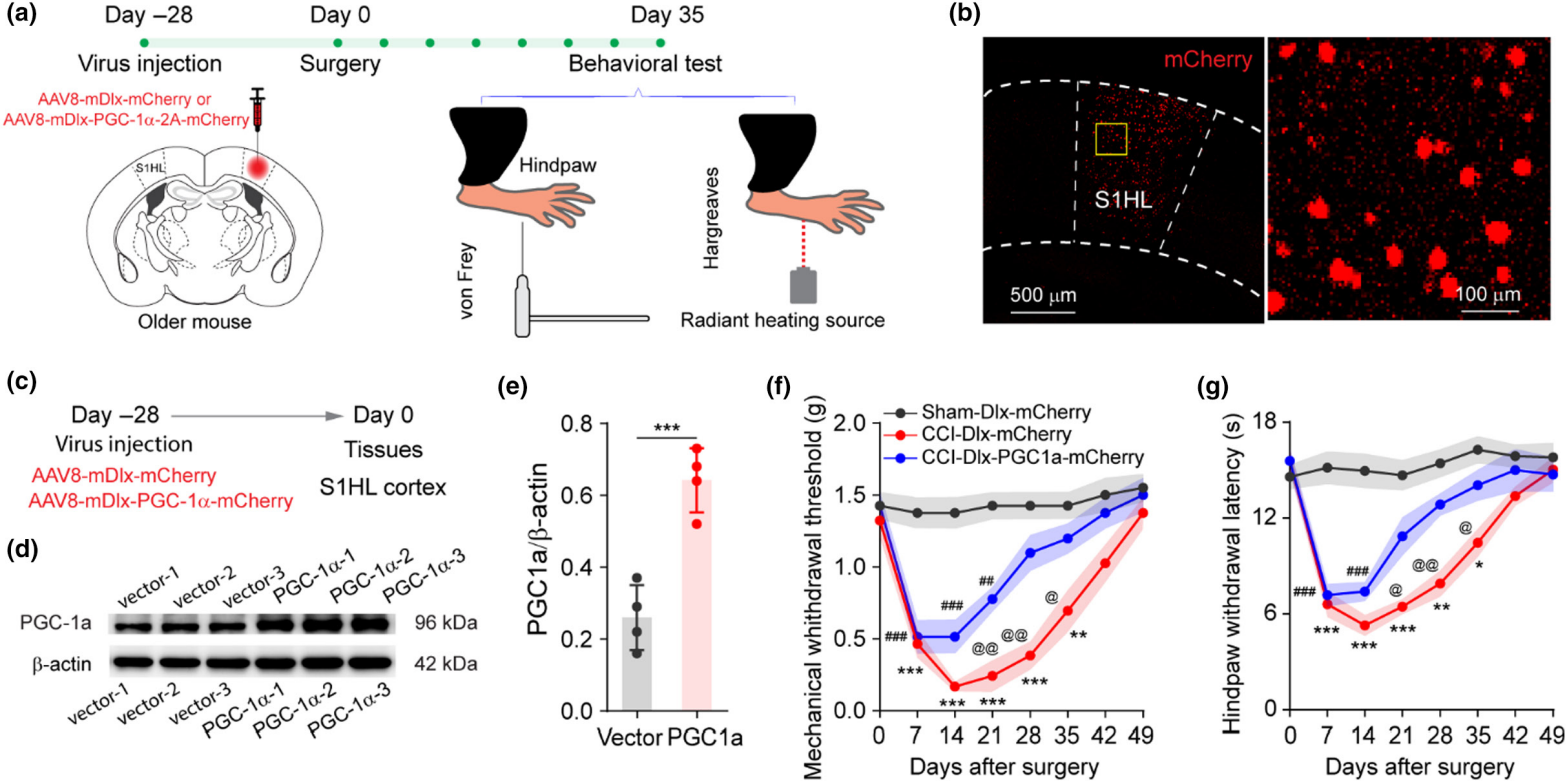
AAV‐mediated overexpression of PGC‐1α in S1HL interneurons alleviates aging‐associated pain chronification. (a) Diagram and sketch depict experiment design. AAV8‐mDlx‐PGC1a‐2A‐mCherry or vector virus was injected into the S1HL of older mice. 4 weeks later the mice either were sacrificed for tissues or received CCI surgery for behavior assessment. (b) Representative image of brain slice showed mCherry expression with boxed region was scanned with a higher magnification objective lens. (c–e) Western blot was performed to verify virus transfection, the brain tissues of S1HL cortex were harvested 4 weeks after virus injection. (c) Diagram of the experiment. (d, e) S1HL PGC‐1a levels. The S1HL PGC‐1a levels were examined using western blot, using β‐Actin as internal control. *N* = 4, data are presented as mean ± SD, two‐tailed unpaired *t* test. ***p < 0.001. (f) Mechanical withdrawal threshold and (g) hindpaw withdrawal latency. *N* = 8, data are presented as mean ± SD, two‐way ANOVA followed by Bonferroni post hoc test was performed for the comparison among groups. Sham‐Dlx‐mCherry versus CCI‐Dlx‐PGC‐1α‐mCherry, *p < 0.05, **p < 0.01, ***p < 0.001; Sham‐Dlx‐mCherry versus CCI‐Dlx‐mCherry, ^##^ *p* < 0.01, ^###^ *p* < 0.001; CCI‐Dlx‐PGC‐1α‐mCherry versus CCI‐Dlx‐mCherry ^@^ *p* < 0.05, ^@@^ *p* < 0.01.

Older mice exhibited lower levels of PGC‐1α in S1 and sustained abnormal S1 neural dynamics (interneuron hypoactivities and excitatory neuron hyperactivities; Figure 3). PGC‐1α has been previously implicated in interneuron function. We speculated that decreased level of PGC‐1α in S1 mediated aging‐related pain chronification. To test this, AAV8‐Dlx‐PGC‐1α‐2A‐mCherry was constructed and injected to the S1HL in older mice (Figure 6a), using AAV8‐Dlx‐mCherry as a control virus (*N* = 4 mice). After 1 month of virus expression in older mice (Figure 6b), S1HL protein western blots showed that AAV8‐Dlx‐PGC‐1α‐2A‐mCherry virus led to significantly increased PGC‐1α levels when compared with vector control (Figure 6c–e). When nociceptive behavior was tested post‐nerve injury (*N* = 8 mice), mice that had received AAV8‐Dlx‐PGC‐1α‐2A‐mCherry were able to resolve nociceptive behavior faster than mice that had received AAV8‐Dlx‐mCherry (Figure 6f,g), suggesting interneuron PGC‐1α was critical for a timely resolution of nociceptive behavior after nerve injury.

### 2.6 Enhanced PGC‐1α expression in S1 interneurons mitigates excitatory neuron hyperactivity in aging‐related pain chronification

**FIGURE 7:**
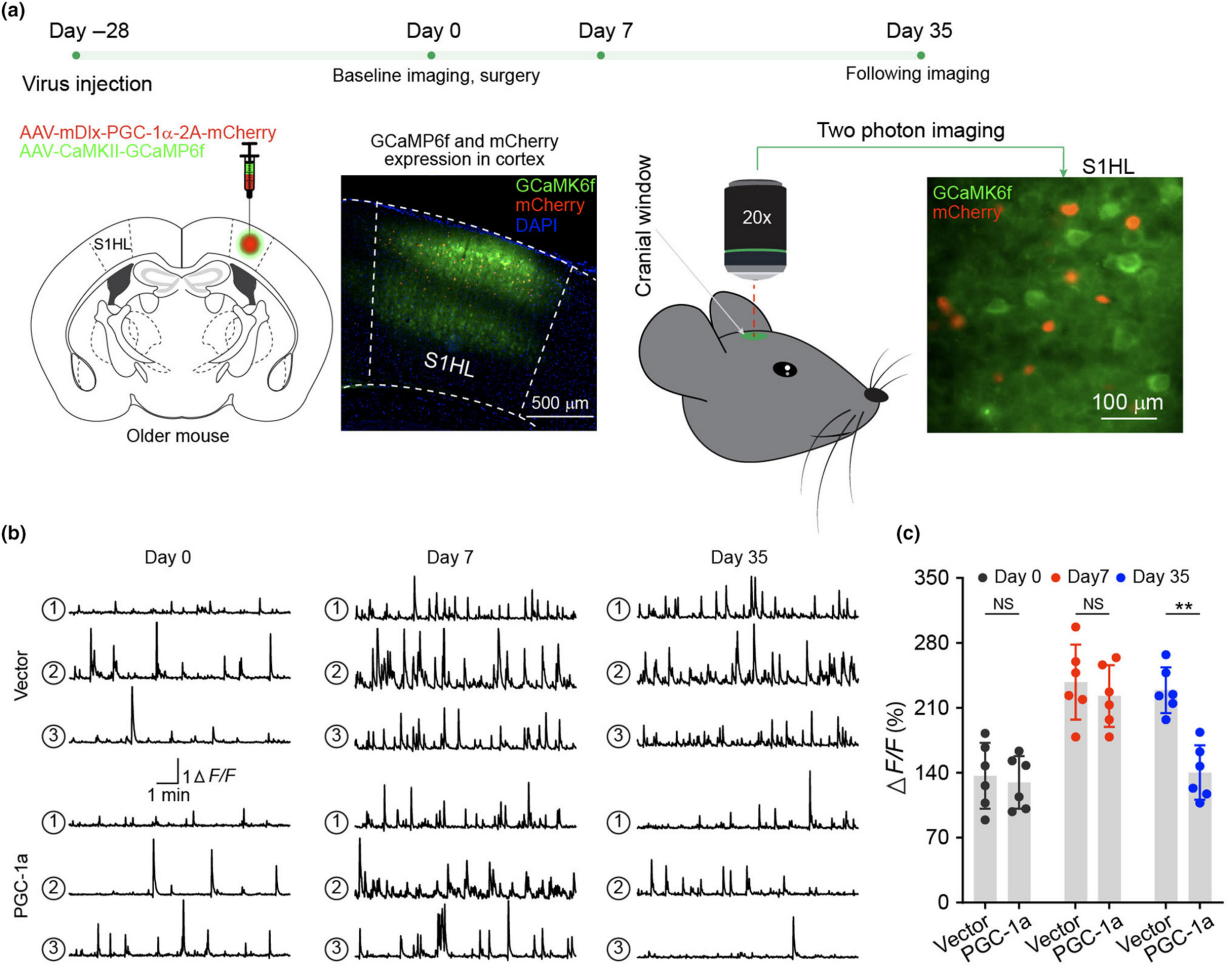
Overexpression of PGC‐1α in S1HL interneurons promotes the resolution of excitatory neuron hyperactivity after CCI in older mice. (a) Diagram and sketch depict experiment design. Mixed viruses of AAV‐mDlx‐PGC‐1a‐2A‐mCherry and AAV‐CaMKII‐GCaMP6f were microinjected into S1HL cortex of older mice. A representative image of brain slice showed viruses expression in S1HL cortex. Intravital two‐photon imaging was performed before CCI surgery at day 0 and following imaging was performed at day 7 and 35 after surgery. (b) Three sample traces of excitatory neuron activity in each group at indicated time points. (c) Integrated calcium signals ΔF/F. *N* = 6, data are presented as mean ± SD, two‐tailed paired *t* test, **p < 0.01. NS: not significant (*p* > 0.05).

To examine S1 neuronal dynamic changes induced by overexpressing PGC‐1α, S1HL was injected AAV8‐Dlx‐PGC‐1α‐2A‐mCherry and AAV9‐CaMKII‐GCaMP6f for interneuron overexpression of PGC‐1α and excitatory neuron calcium imaging (Figure 7a), respectively. In older mice that received AAV8‐Dlx‐mCherry vector control, nerve injury led to sustained excitatory neuron hyperactivities (*N* = 6). In contrast, in older mice that received AAV8‐Dlx‐PGC‐1α‐2A‐mCherry, interneuron overexpression of PGC‐1α led to resolution of excitatory neuron hyperactivities by day 35 (Figure 7b,c), coinciding with resolution of nociceptive behavior shown in Figure 6.

## 3 DISCUSSION

Chronic pain has high prevalence in older age group, posing significant societal and medical burdens. Whether aging‐associated changes in the central nervous system predispose older individuals to chronic pain is unknown. In this report, using a mouse model of nerve injury‐induced nociceptive pain, older mice displayed significantly prolonged nociceptive behavior than mature adults, consistent with aging‐associated pain chronification. Using two‐color intravital two‐photon calcium imaging to simultaneously investigate excitatory neuron and interneuron activities in the S1, aging‐associated pain chronification was accompanied by excitatory neuron hyperactivity and interneuron hypoactivity. Mechanistically, S1 in older mice had lower levels of PGC‐1α than mature adults. PGC‐1α has been shown to be critical for interneuron functional integrity (Lucas et al., 2010, 2014; Vanaveski et al., 2021). Chemogenetic inhibition of S1 interneurons precipitated nociceptive behavior in naïve mice, whereas chemogenetic activation of S1 interneurons alleviated nociceptive behavior in older mice following nerve injury. More importantly, overexpression of PGC‐1α in S1 interneurons of older mice alleviated aging‐associated pain chronification and dampened S1 excitatory neuron hyperactivity. As such, these results support a critical role for PGC‐1α in aging‐associated pain chronification.

One potential limitation of our study is that we did not examine spinal and dorsal root ganglia (DRG) mechanisms. Studies have demonstrated the significance of spinal cord and DRG in pain modulation, as well as cortical mechanisms in pain perception. For example, activation of cortical somatostatin interneurons has been shown to prevent neuropathic pain development in animal models (Cichon et al., 2017). Additionally, the importance of DRG and spinal cord alterations in pain processing has been elegantly examined in previous studies, including in Petitjean et al. (2015). Moreover, recent research has highlighted the intricate interactions between cortical and spinal contributions to pain processing. Corticospinal projections have been implicated in modulating touch and tactile neuropathic pain sensitivity (Liu et al., 2018), providing a framework to connect DRG/spinal cord and cortical modulations. In our recent study, we demonstrated that while cortical interneuron modulation influenced nociceptive behavior in a mouse model of trigeminal neuralgia, peripheral decompression led to dampened pain‐related cortical neural dynamics and alleviation of nociceptive behavior (Ding, Yang, et al., 2023). These findings underscore the complex interplay between cortical and spinal mechanisms in pain modulation. Another potential limitation is that we employed reflex‐based nociceptive behavior, while affective components of pain were not included in the study. Recent studies have highlighted the influence of S1 manipulations on both sensory and affective pain responses (Ziegler et al., 2023). Alterations in neural activity within the S1 cortex have been associated not only with changes in sensory thresholds but also with affective aspects of pain processing. Future studies incorporating affective behavioral read‐outs alongside sensory assessments will provide a more comprehensive understanding of our results.

PGC‐1α is known to be ubiquitously expressed, with particularly high levels observed in tissues with high metabolic demands, such as skeletal muscle, heart, and brain regions like the hippocampus and cortex (Sahin et al., 2011; Wang et al., 2021). This widespread expression underscores its importance in coordinating metabolic homeostasis and cellular energy production throughout the body. Previous research has shown that alterations in PGC‐1α levels can impact various physiological processes, including mitochondrial biogenesis, oxidative metabolism, glucose homeostasis, and cellular responses to stress (Sahin et al., 2011; Zhao et al., 2008). As a mitochondrial biogenesis master regulator, PGC‐1α has been found to be critical for neuronal function. For example, PGC‐1α knockout (−/−) mice displayed severe neurological symptoms and died prematurely (St‐Pierre et al., 2006). PGC‐1α expression is concentrated in GABAergic interneurons, and it provides a direct link between external physiological stimuli and the regulation of mitochondrial biogenesis (Lucas et al., 2014). Interneurons are indispensable in modulating neural network function and are implicated in learning, memory, and sensory processing (B. Liu et al., 2022; Sun et al., 2020; Tremblay et al., 2016; Zhou et al., 2023). Our results showed that older mice had decreased S1 expression of PGC‐1α and prolonged interneuron hypoactivities after sciatic nerve injury. Correction of aging‐related PGC‐1α decrease using AAV‐mediated PGC‐1α expression alleviated aging‐associated pain chronification, supporting PGC‐1α as a potential therapeutic target to reduce the burden of chronic pain in older individuals. Previously, PGC‐1α has been shown as a promising candidate to ameliorate aging‐related musculoskeletal deterioration, cardiomyopathy, and postoperative delirium (Kang & Li Ji, 2012; Zhou et al., 2023; Zhu et al., 2019). Both pharmaceutical and non‐pharmaceutical interventions could modulate PGC‐1α expression. For example, anti‐diabetic drug metformin has been shown to increase PGC‐1α expression (Aatsinki et al., 2014; Miao, Zhou, et al., 2020). The health‐promoting effects of exercise and caloric restriction have been proposed to depend, at least partially, on PGC‐1α (Handschin & Spiegelman, 2008; Lettieri Barbato et al., 2012). More recently, gut microbiome metabolite, indole‐3 propionic acid, has been shown to modulate PGC‐1α expression (Zhou et al., 2023).

Interneurons provide fundamental mechanisms for information processing during sensory perception and memory formation through orchestrating neuronal network activities. From a neuroenergetic standpoint, inhibitory interneurons, including parvalbumin neurons, have high metabolomic demand and are highly dependent on mitochondrial function (Kann et al., 2014). As a result, they are susceptible to oxidative stress, neuroinflammation, and other insults (Crapser et al., 2020; Powell et al., 2012; Roque et al., 2023). As such, we speculate that aging‐related decrease of PGC‐1α might predispose interneurons to dysfunction. Notably, mitochondrial dysfunction has been recognized as one of the hallmarks of aging (Lopez‐Otin et al., 2023). Our results showed that in mature adult mice, interneuron hypoactivity was restored to baseline by day 35 post sciatic nerve injury, along with resolution of nociceptive behavior. In contrast, in older mice, interneuron hypoactivity failed to restore during the same time frame, which led to prolonged nociceptive behavior. Thus, improving mitochondrial health might promote healthy aging and alleviate aging‐associated comorbidities.

Intravital two‐photon calcium imaging has greatly accelerated the exploration of neuronal activities in physiology and pathological conditions (Svoboda & Yasuda, 2006). Using a red variant of genetically encoded calcium indicator (Dana et al., 2016) in conjunction with a green color calcium sensor, we could simultaneously image excitatory neuron and interneuron activities in the setting of sciatic nerve injury. Our results support that aging‐related changes in the central nervous system predispose older individuals to chronic pain, which could be targeted to treat aging‐related pain chronification and promote successful aging.

## 4 MATERIALS AND METHODS

### 4.1 Animals

All experiments in this study are in accordance with the National Institutes of Health guidelines/regulations and approved by Massachusetts General Hospital Institutional Animal Care and Use Committee. Adult male/female C57BL/6 mice between 12 and 16 weeks of age and older male/female C57BL/6 mice between 17 and 18 months of age at the start of the experiment were used. Efforts were made to minimize the number of animals used in this study. Mice were purchased from the Jackson Laboratory (USA) and housed in a temperature‐controlled colony room on a 24‐h light cycle (lights on at 7 am; lights off at 7 pm), such that behavioral testing was performed during the light phase of the cycle. Mice were well maintained on ad libitum food and water. Additionally, the animal care and monitoring procedures followed the ARRIVE guidelines for transparent reporting. Procedures to mitigate pain, suffering, and distress were detailed in the experimental protocols. Any anticipated or unforeseen adverse events were documented. Humane endpoints, including monitored signs and their frequency, were established for the study. C21 (Tocris, catalog 5548) was intraperitoneally administered at 1 mg/kg.

### 4.2 Chronic constriction injury of sciatic nerve surgery (CCI)

Mice were anesthetized with oxygenated isoflurane (2.5% for induction; 1.5%–2% for maintenance) using a customized nose cone. After the right side of lower extremity was antiseptically prepared with 10% povidone‐iodine solution (Medline Industries Inc., Northfield, IL), the right side sciatic nerve was dissected following 1% lidocaine infiltration. CCI was produced by loosely ligating the exposed sciatic nerve referring to the method of Bennett and Xie (Bennett & Xie, 1988; Zhou et al., 2023) using four 6–0 chromic gut sutures with 2 mm apart. Sham mice were made following the same surgical process except for never‐ligation. The skin was opposed with three 6–0 vicryl sutures (Ethicon, Somerville, NJ).

### 4.3 Mechanical withdrawal threshold

All behavioral tests were conducted by an investigator who was blinded to the animal groups and surgeries. von Frey test was used to assess mechanical withdrawal threshold following the method as previously described (Shen et al., 2017). Briefly, mice were habituated for 30 min for 3 consecutive days before baseline behavior assessment. On the testing day, mice were placed on an elevated mesh platform and covered with a transparent Plexiglas chamber (10 × 10 × 12 cm). After 10–20 min of acclimation, withdrawal responses to punctate mechanical stimuli were tested using the von Frey filaments (Figure 1b). Each fiber was applied five times to the plantar aspect of the left hind paw for 1 s with a 10‐s interval between each stimulation. The test started with the force of 0.04 grams and continued ascending order up to 2 grams (cutoff). The mechanical withdrawal threshold was defined as the force at which withdrawal occurred at least three out of five applications, and two grams was recorded as the threshold if less than three positive responses to all filaments.

### 4.4 Hargreaves test

Thermal hyperalgesia to heat stimulation was evaluated following the procedure as previously described (Yang et al., 2022). Briefly, mice were habituated 30 min for three consecutive days before baseline behavior assessment. On the testing day, mice were placed on a preheated glass platform (~28°C) and covered with transparent Plexiglas cubicles for ~10 min. Remove mice drops when necessary. Using a radiant heat source underneath the glass and focus the light beam to the middle of the hindpaw underwent CCI surgery (Figure 1b). Paw withdrawal latency was defined as the time duration (seconds) from the initiation of heat exposure to the hindpaw withdrawal. To avoid tissues burning, 20 s was set as the cut‐off time.

### 4.5 Virus injection and cranial window implantation

The procedures were performed as previously described (Ding, Fischer, et al., 2023; Ding, Yang, et al., 2023). Briefly, mice were anesthetized with oxygenated isoflurane (3% for induction and 1.5% for maintenance) while the respiratory rate was closely monitored. To avoid corneal dehydration, eye lubricant was applied to moisten the eyes of mice. To minimize perioperative pain, Ketorolac tromethamine (Althenex, Schaumburg, IL USA) was administrated intraperitoneally (5 mg/kg) 30 min before surgery and every 12 h after surgery for 2 consecutive days. The dorsal fur between outer canthus and concha level was shaved, and the mouse was mounted onto a stereotactic frame with ear bars. Secure the front teeth onto the incisor bar of the frame. Mouse snout was then covered by a mouse anesthetic mask delivering oxygenated anesthetic gases. The skin was prepared with Povidone‐Iodine Solution (Aplicare, Inc., Neriden, CT, USA) followed by 70% alcohol swab (BD, Franklin Lakes, USA). After lidocaine infiltration (1%, 0.2 mL), the skin overlying the dorsal skull was removed. Using a curved scalpel blade (#12) to remove a 3 × 3 mm bone flip covering the left side of somatosensory cortex (S1HL) with dura intact. 1 μL of AAV9‐CamKII‐GCaMP6f (Addgene, Plasmid #100834), AAV8‐hDlx‐GCamP6f (Vigene), AAV8‐CaMKII‐jRGECO1a (Canadian Neurophotonics Platform Viral Vector Core Facility, RRID:SCR_016477), AAV9‐hDlx‐Gi DREADD‐dTomato (Addgene, plasmid #83896), AAV9‐hDlx‐GqDreadd‐dTomoto (Addgene, plasmid #83897) and AAV8‐hDlx‐dTomato (from Qian Chen) was microinjected into left S1HL cortex (Bregma −0.95 mm, lateral 1.5 mm, depth 200–400 nm) using a Nanoject (Drummond Scientific Company, Broomall, USA) with a glass needle in ~100 μm diameter. Immediately after the virus injection, a 3 mm rounded coverslip overlayed by a 5 mm rounded cover slip was placed in the cranial window and cemented using adhesive luting cement (C&B‐Metabond, #171032, Parkell, Edgewood, NY, USA). A customized metal bar was attached for fixation during intravital two‐photon imaging.

### 4.6 In vivo transcranial two‐photon imaging

After 28 days of recovery and viral transfection, mice were acclimated under a two‐photon microscope with head restrained 30 min for three consecutive days. The baseline imaging was acquired 1 day before CCI or sham surgery using the two‐photon microscope equipped with a Mai Tai laser (Spectra Physics, KMC 100). The wavelength was set at 910 nm, and the average laser power output was ∼25 mV during imaging. A 20×, 1.0 NA water immersion objective (Olympus, Japan) was used for imaging acquisition, and Prairie View Software was used for data collection as previously described (Ding, Fischer, et al., 2023; Yang et al., 2022). Following imaging was acquired at desired time points. All images were acquired at frame rate of 6–12 Hz in awake status without anesthesia.

### 4.7 Calcium imaging data analysis

Imaging data was corrected for motion between frames using the NoRMCorre software package (Pnevmatikakis & Giovannucci, 2017). Neuron selection was carried out subsequently using customized transcription in Matlab (Mathworks). Calcium fluorescence signals of each individual neuron were extracted from the corrected image series. The signal for each neuron was corrected for background fluorescence changes by subtracting the fluorescence changes from the immediate surrounding. Each neuron's activity time course was then quantified using the formula ∆*F* = (*F* – *F*_*0*_)/*F*_*0*_, where *F* is the fluorescence signal at a given frame and *F*_*0*_ was calculated from a sliding window of +/− 30 s around the frame. Finally, baseline correction was carried out by fitting a linear function (Matlab function robustfit) to the lowpass‐filtered (cutoff: 0.3 Hz) signal. A deconvolution algorithm (Fast online deconvolution of calcium imaging data) was applied to detect transients (Friedrich et al., 2017). The start and end of transients were detected when the model was above 0.1 as previously described (Ding, Fischer, et al., 2023; Ding, Yang, et al., 2023). All codes are available at https://github.com/harnett/Shiqiananalysis.

### 4.8 Western blot

Fresh tissues were collected immediately after the animals were sacrificed. Protein lysates were obtained from cortex, and the protein concentrations were measured using a Bradford Protein Assay kit (ab102535). The absorbance at 595 nm was read in a microplate reader and is directly proportional to the amount of protein bound. The exact protein concentration of the sample is determined by interpolation from a standard curve made by measuring the absorbance of a dilution series of protein standards of known concentrations within the linear response range of the assay. Equal amount of protein (30 μg) samples were separated through electrophoresis and subsequently transferred to a polyvinylidene difluoride (PVDF) membrane. Blocking the membrane with 5% skim milk at room temperature (RT) for 1 h. Primary antibody of anti‐PGC1a (1:500, rabbit polyclonal; Abcam) or anti‐β‐actin (1:5000, mouse monoclonal; Sigma) were incubated at 4°C overnight. Wash the membrane with PBST 10 min three times. HRP‐conjugated secondary antibody (1:5000, Jackson ImmunoResearch) was incubated for 1 h at RT. Washing the membrane with PBST again was followed by protein bands imaging using a chemiluminescence equipment, and the density of each band was analyzed using ImageJ software (National Institutes of Health, USA).

### 4.9 Fluorescence/immunofluorescence imaging

Mice were anesthetized and transcardially perfused with ice‐cold PBS followed by 4% paraformaldehyde. Brain samples were placed in 4% PFA and stored at 4°C for 2 days. Washing the brain samples using 1 × PBS, and the brain hemispheres were sliced to 50 μm slices using a Leica vibratome (VT 1000s). Free‐floating sections throughout the S1HL cortex were rinsed in PBS and then incubated with primary antibody of polyclonal rabbit anti‐GABA (1:500, Sigma‐Aldrich) in blocking buffer (0.05% triton +6% BSA) at 4°C overnight. Slices were then washed and incubated with polyclonal goat anti‐rabbit Alexa488 (1:2000; Invitrogen) for 1 h at room temperature. Images were acquired using a Nikon AX confocal microscope (NIKON, Japan), and images were taken in ×4 and × 20 objectives. Data were analyzed using ImageJ (NIH open‐source software).

### 4.10 Statistical analyses

All behavioral data in line charts were presented as mean ± SEM and mean ± SD in bar charts. According to our previous studies on pain behavior in mice, sufficient power to detect a significant effect should be achieved using at least eight mice per group for the behavioral assessments, four mice per group for immunofluorescence analysis, at least three mice per group for western blot, and 4–6 mice for intravital imaging. Based on mechanical withdrawal threshold and thermal paw withdrawal latency tests, the difference in pain behaviors was analyzed using a repeated‐measures two‐way analysis of variance (ANOVA). Post‐hoc comparisons with Bonferroni corrections were used to compare the thresholds across groups at indicated time points. *p* < 0.05 was defined as statistically significant, and significance testing was two‐tailed in a two‐group comparison. For Bonferroni corrections, the adjusted *p*‐values, calculated via dividing *p*‐values by sample size, are reported. Statistical analysis was conducted using GraphPad Prism software (version 8.0).

## AUTHOR CONTRIBUTIONS

S. S. and W. D. planned and designed the experiments; L. Y., W. D., and J. M. coordinated all the experiments; X. W. conducted pain model, virus injection and behavioral test. L.Y. performed western blot and immunofluorescent staining; Z. L., C. G., K. J., A. L., N. F. R., K. C., I. F. and A. C. assisted data processing and parts of work including surgery, behavioral testing and western blot; W. D. and Q. C. carried out imaging experiment; L. Y. and W. D. analyzed the data and presented the figures. W. D. and S. S. drafted the manuscript.

## FUNDING INFORMATION

Shiqian Shen lab received support from NIH R61NS116423, NIH R35GM128692, NIH R01 AG 070141, R01 AG082975‐01A1, NIH R03 AG067947, and NIH R61 NS126029. Part of the study was supported by NSF EAGER 2334666. Weihua Ding received support from the Borsook Project.

## CONFLICT OF INTEREST STATEMENT

The authors have declared that no conflict of interest exists.

## Supporting information


Figures S1–S4.

## Data Availability

All the data that support the findings of this study are available from the corresponding authors upon reasonable request.
