# Supplementary material for: Aging‐associated decrease of PGC‐1α promotes pain chronification
Source: Aging Cell. 2024 May 17;23(8):e14177. doi: 10.1111/acel.14177 (PMC11320346; doi:10.1111/acel.14177)
Supplement: Supplementary file 1 — Figures S1–S4. [file ACEL-23-e14177-s001.docx]

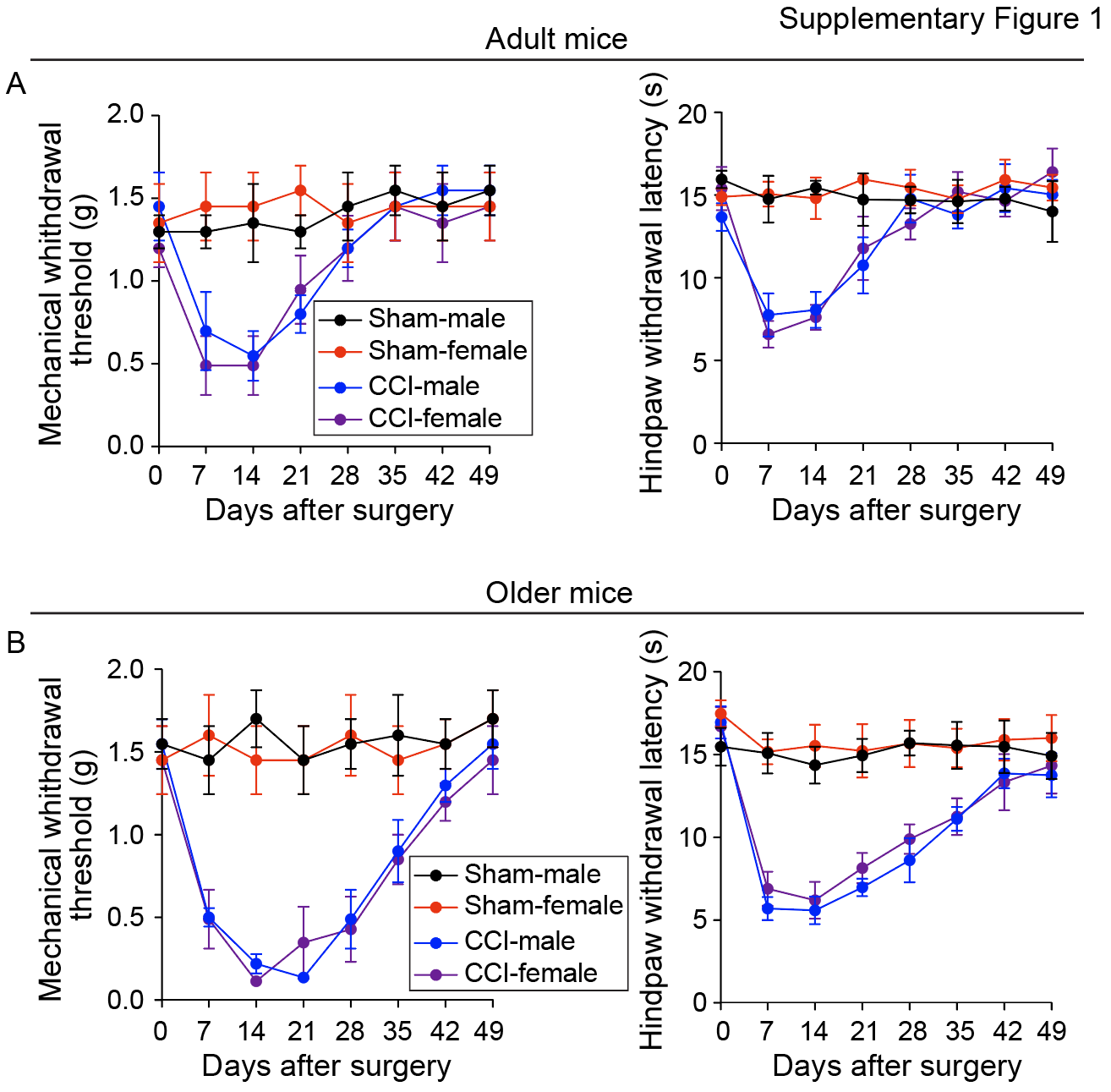


**Supplementary figure 1.** **Behavioral changes in response to CCI in male and female mice**. After baseline test, mice received either sham or CCI surgery which was followed by behavioral tests at indicated timepoints. **A**) Mechanical withdrawal threshold and hindpaw withdrawal latency in adult mice. **B**) Mechanical withdrawal threshold and hindpaw withdrawal latency in older mice. N = 4 mice, two-way ANOVA test followed by Bonferroni post hoc analysis was conducted to ascertain differences among groups. The statistical analysis indicated no significant difference between adult or older male and female mice across all time points.


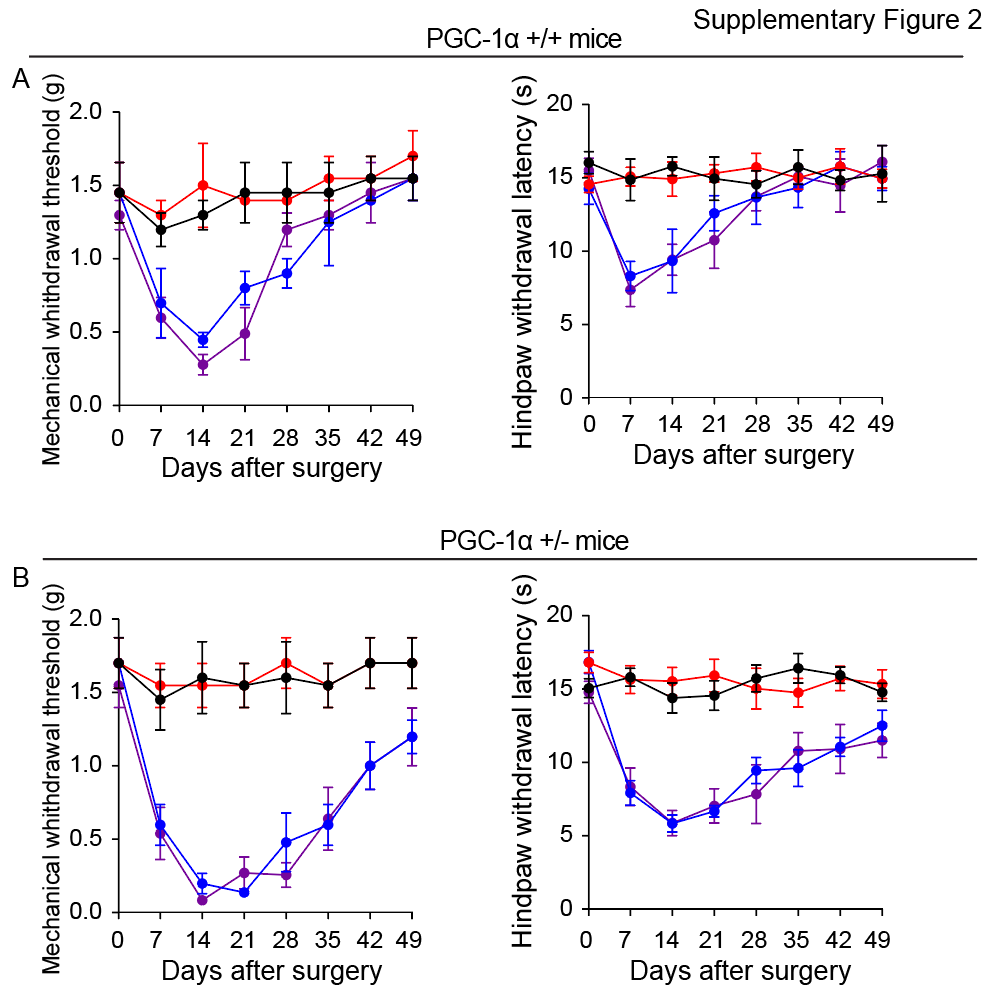


**Supplementary figure 2. Behavioral changes in response to CCI in male and female PGC-1α +/-** **mice**. After baseline test, mice received either sham or CCI surgery which was followed by behavioral tests at indicated timepoints. **A**) Mechanical withdrawal threshold and hindpaw withdrawal latency in PGC-1α +/+ mice. **B**) Mechanical withdrawal threshold and hindpaw withdrawal latency in PGC-1α +/- mice. N = 4 mice, two-way ANOVA test followed by Bonferroni post hoc analysis was conducted to ascertain differences among groups. The statistical analysis revealed no significant difference between PGC-1α +/+ or PGC-1α +/- male and female mice across all time points.


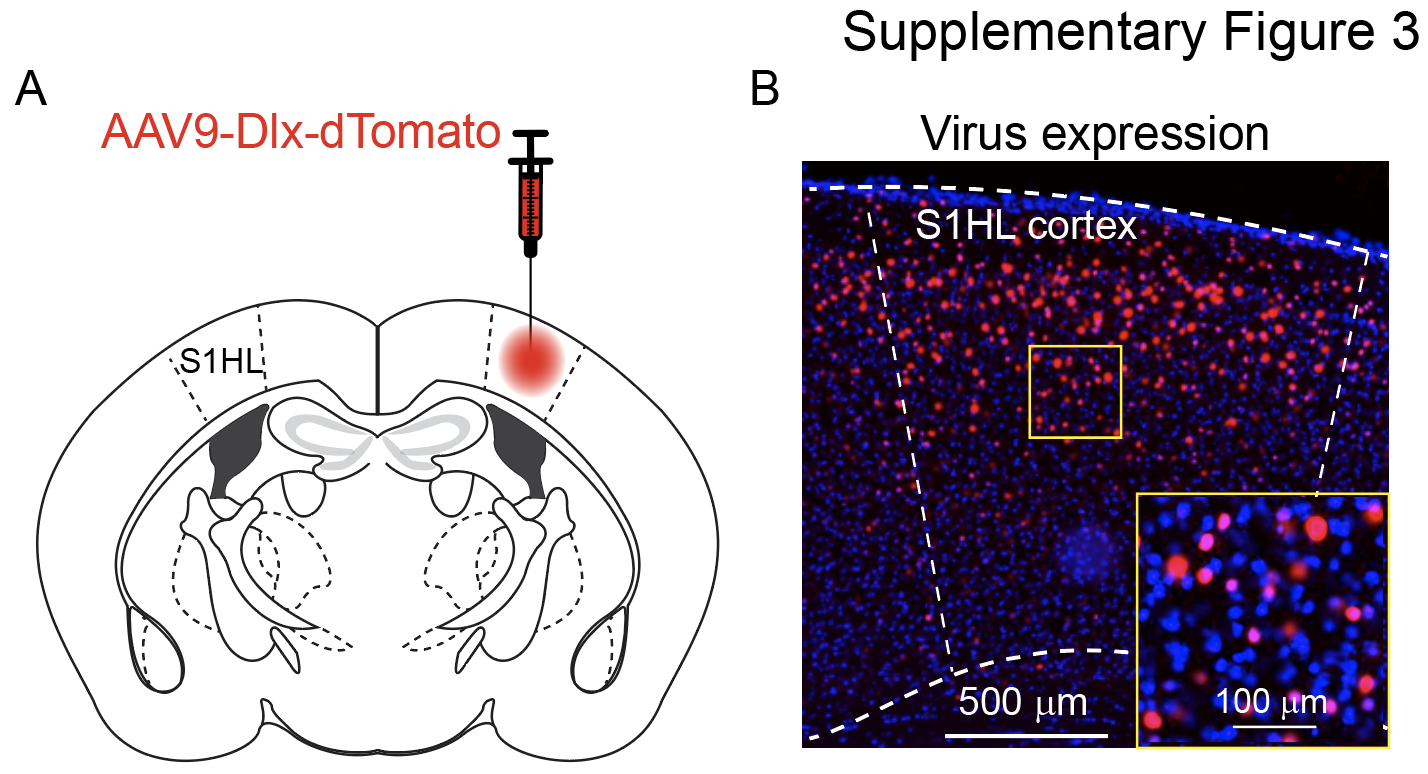


**Supplementary figure 3.** **Control virus AAV9-Dlx-dTomato expression**. **A**) Schematic Representation of AAV9-Dlx-dTomato Virus Injection into the S1HL Cortex. Animals were allowed a resting period of four weeks for virus transduction. **B**) Representative Image Demonstrating AAV9-Dlx-dTomato Virus Expression.


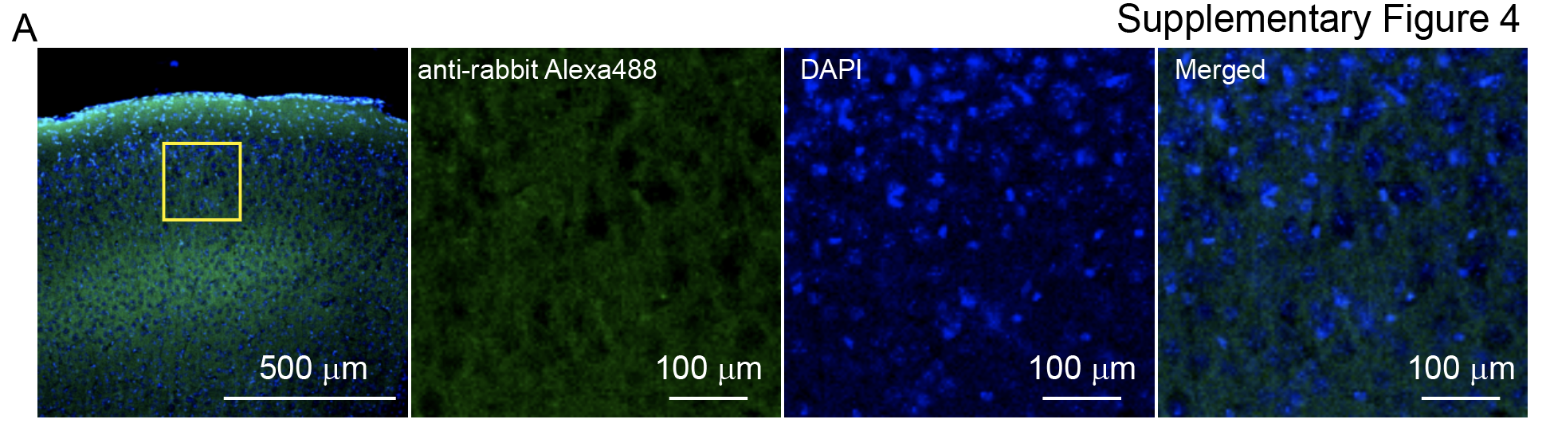
**Supplementary figure 4. Negative control images for GABA staining**. **A)** Negative control images using anti-rabbit Alexa488 as the secondary antibody without the primary antibody of rabbit anti-GABA. The boxed region in the S1HL cortex was scanned using a 20x objective.
